# Automatic segmentation of dura for quantitative analysis of lumbar stenosis: A deep learning study with 518 CT myelograms

**DOI:** 10.1002/acm2.14378

**Published:** 2024-05-10

**Authors:** Guoxin Fan, Yufeng Li, Dongdong Wang, Jianjin Zhang, Xiaokang Du, Huaqing Liu, Xiang Liao

**Affiliations:** ^1^ Department of Pain Medicine Huazhong University of Science and Technology Union Shenzhen Hospital Shenzhen China; ^2^ Department of Spine Surgery Third Affiliated Hospital Sun Yat‐Sen University Guangzhou China; ^3^ Department of Sports Medicine Eighth Affiliated Hospital Sun Yat‐Sen University Shenzhen China; ^4^ Department of Orthopaedics Putuo People's Hospital School of Medicine, Tongji University Shanghai China; ^5^ Department of Orthopedics The People's Hospital of Wenshang County Wenshang Shandong China; ^6^ Artificial Intelligence Innovation Center Research Institute of Tsinghua PearlRiverDelta Guangzhou China

**Keywords:** CT myelograms, deep learning, dural sac, lumbar stenosis, semantic segmentation

## Abstract

**Background:**

The diagnosis of lumbar spinal stenosis (LSS) can be challenging because radicular pain is not often present in the culprit‐level localization. Accurate segmentation and quantitative analysis of the lumbar dura on radiographic images are key to the accurate differential diagnosis of LSS. The aim of this study is to develop an automatic dura‐contouring tool for radiographic quantification on computed tomography myelogram (CTM) for patients with LSS.

**Methods:**

A total of 518 CTM cases with or without lumbar stenosis were included in this study. A deep learning (DL) segmentation algorithm 3‐dimensional (3D) U‐Net was deployed. A total of 210 labeled cases were used to develop the dura‐contouring tool, with the ratio of the training, independent testing, and external validation datasets being 150:30:30. The Dice score (DCS) was the primary measure to evaluate the segmentation performance of the 3D U‐Net, which was subsequently developed as the dura‐contouring tool to segment another unlabeled 308 CTM cases with LSS. Automatic masks of 446 slices on the stenotic levels were then meticulously reviewed and revised by human experts, and the cross‐sectional area (CSA) of the dura was compared.

**Results:**

The mean DCS of the 3D U‐Net were 0.905 ± 0.080, 0.933 ± 0.018, and 0.928 ± 0.034 in the five‐fold cross‐validation, the independent testing, and the external validation datasets, respectively. The segmentation performance of the dura‐contouring tool was also comparable to that of the second observer (the human expert). With the dura‐contouring tool, only 59.0% (263/446) of the automatic masks of the stenotic slices needed to be revised. In the revised cases, there were no significant differences in the dura CSA between automatic masks and corresponding revised masks (*p *= 0.652). Additionally, a strong correlation of dura CSA was found between the automatic masks and corresponding revised masks (*r* = 0.805).

**Conclusions:**

A dura‐contouring tool was developed that could automatically segment the dural sac on CTM, and it demonstrated high accuracy and generalization ability. Additionally, the dura‐contouring tool has the potential to be applied in patients with LSS because it facilitates the quantification of the dural CSA on stenotic slices.

**Abbreviations**
3D: 3‐dimensional
AI: artificial intelligence
CSA: cross‐sectional area
CT: computed tomography
CTM: computed tomography myelogram
DCS: dice score
DL: deep learning
HD: Hausdorff distance
LSS: lumbar spinal stenosis
MRI: magnetic resonance imaging
ROI: regions of interest

## 1 BACKGROUND

Lumbar spinal stenosis (LSS) is one of the main causes of chronic pain and disability, affecting approximately 103 million people worldwide.^1^ The degenerative changes in the discs, ligamenta flava, and facet joints lead to the narrowing of the spaces around the neurovascular structure of the spine, resulting in substantial pain, impaired ambulation, and disability.^2^ Typical symptoms of LSS include intermittent claudication and lower back pain, with or without accompanying radicular pain. The diagnosis of LSS can be a challenging task,^3^, ^4^, ^5^, ^6^ especially for the localization of the culprit levels because radicular pain is not often present. Accurate identification of the culprit levels is key to effective pain management and precision surgery. The intervention costs of LSS greatly burden society, and this burden continues to increase as the global population ages.^7^, ^8^ Thus, any progress in neuroimaging techniques that enables the differential diagnosis of LSS offers substantial benefits to patients and society.

The quantitative analysis of radiologic images is often used to diagnose LSS. Imaging modalities, including computed tomography (CT), magnetic resonance imaging (MRI), and CT myelography (CTM), are extensively used for assessing LSS. The North American Spine Society emphasized the significance of imaging tests in their guidelines, yet no radiological criteria were provided.^9^ Existing literature also reported remarkable variability in the radiologic criteria.^3^ Hence, researchers have attempted to quantify the radiological features of LSS. Among the numerous proposed radiological biomarkers, the cross‐sectional area (CSA) of the dural sac has been frequently used to assess central LSS.^10^, ^11^, ^12^ The first step of CSA quantification is to delineate the dural boundaries on medical images.^13^ However, the manual delineation of the dural sac on medical images is cumbersome and inconsistent among different observers.^13^

The development of artificial intelligence (AI) has facilitated the contouring of regions of interest (ROIs) on medical images, ensuring a process that is both efficient and consistent.^14^ Deep learning (DL) is a popular AI technique that enables more feature fitting from a large dataset to develop an accurate automatic contouring tool, eliminating interobserver variability and facilitating large‐throughput analysis pipelines.^15^ An AI contouring tool can greatly contribute to the quantitative image analysis of certain diseases. For example, the spinal cord toolbox is an automatic ROI contouring tool with quantitative analysis for the cervical dura or spinal cord on MRI, which has been implemented in many studies^16^, ^17^ and widely acknowledged in research communities as a reliable method to analyze images of cervical spondylotic myelopathy and spinal cord injury.

Similarly, many studies have endeavored to achieve the automatic segmentation of the lumbar dura on medical images.^13^, ^18^ For the purpose of automatic segmentation, 3‐dimensional (3D) U‐Net is the baseline DL algorithm capable of precisely delineating ROIs, including the dural sac, within CT and MRI images.^19^, ^20^ Previous studies also verified that automatic segmentation of the dural sac on MR images can be useful for establishing objective criteria for diagnosing and assessing LSS.^21^, ^22^ However, to the best of our knowledge, no studies have yet applied these DL models for automatic segmentation of the lumbar dura on CTM images. Thus, the aim of the present study was to develop a DL‐based contouring tool for the automatic segmentation and quantitative analysis of the lumbar dura on CTM images. We present the following article in accordance with the TRIPOD reporting checklist.

## 2 MATERIALS AND METHODS

### 2.1 Data collection

This study was conducted in accordance with the Declaration of Helsinki (as revised in 2013). The institutional ethical committees of our institutions approved the study (No. [2020]02‐252‐01; KY‐2022‐031‐01) and written informed consent for this retrospective study was waived due to the retrospective nature of the study. Medical records and radiologic data of patients who underwent CTM scans in our hospital were retrospectively collected between January 2014 and December 2021. The inclusion criterion was patients who underwent CTM scans, with or without LSS. The exclusion criteria were as follows: (1) patients complicated with an intraspinal tumor, (2) patients with a congenital spinal malformation (e.g., tethered cord, diplomyelia), and (3) patients who underwent an unsuccessful contrast injection (e.g., epidural distribution of the contrast). We also recorded the baseline characteristics of these patients, including age (years), gender (male, female), and radiologic stenotic levels (L1/L2 to L5/S1). At the time of the study, there was no standard for estimating the number of samples required for a machine learning study.^23^ Usually, a minimum of 50 cases should be included when developing a DL model of semantic segmentation on lumbar CT.^24^, ^25^ The more data that are included, the more convincing the interpretation of the results becomes. Thus, a total of 518 CTM cases were included in the study. Data from 180 CTM cases were used to develop and test the AI model, while data from another 30 CTM cases were used for external validation. The major difference of the external CTM data was reconstructed with the bone algorithm from 30 independent patients, while other CTM data were reconstructed with a soft‐tissue algorithm from other patients. The two kinds of CT reconstruction algorithms generate CT images with different textures that are not affected by variations in window width and window level (see Figure S1). In this study, the CTM images were acquired by CT equipment including SIEMENS Drive CT and Philips series CT. The scan settings were as follows: slice thickness of 1−5 mm (median 3 mm), 120−135 kV (median 120 kV), and 60−200 mA (median 200 mA).

### 2.2 Dura labeling

**FIGURE 1:**
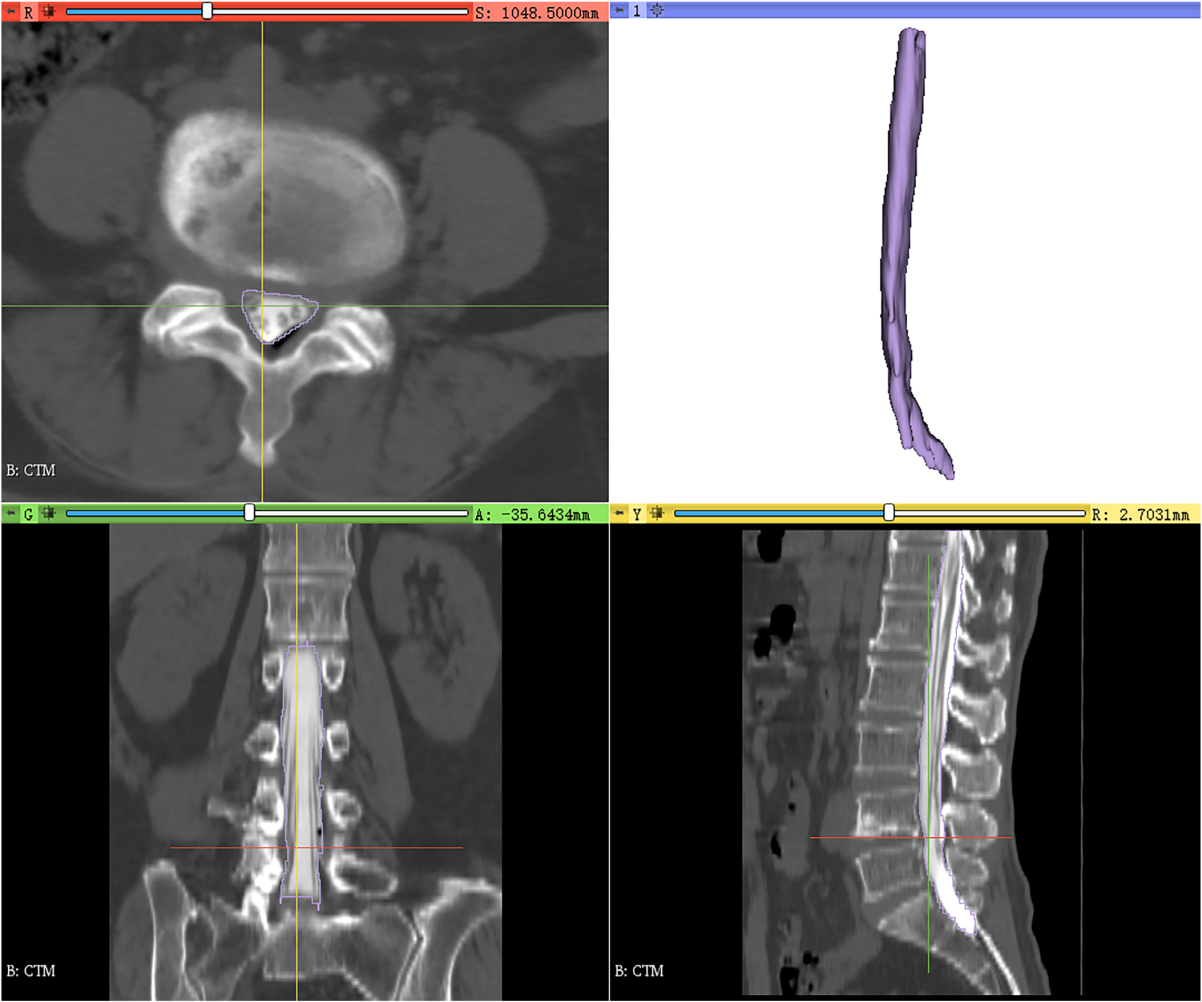
The manual segmentation of the dura. The axial, coronal, sagittal, and 3D views of the segmented dural sac of the lumbar spine. 3D, 3‐dimensional.

The data from 518 CTM cases included 210 manually labeled and fully segmented CTM cases and 308 unsegmented CTM cases. The manually labeled data were initially segmented by a human expert using Slicer 5.0.3 software^26^ (http://www.slicer.org) (Figure 1) and then reviewed by another two experts. All the experts had at least 5 years of experience in reading lumbar CTMs, along with at least 1 year of experience in operating the 3D Slicer. Before labeling the dural sac, these experts received the same relevant training. Any disagreements regarding the segmented area were voted on and revised by these three experts, and the post‐review expert segmentation was regarded as the ground truth of the dura masks. These fully segmented CTM images were used to train, validate, and test the DL algorithms.

### 2.3 Development of DL models

**FIGURE 2:**
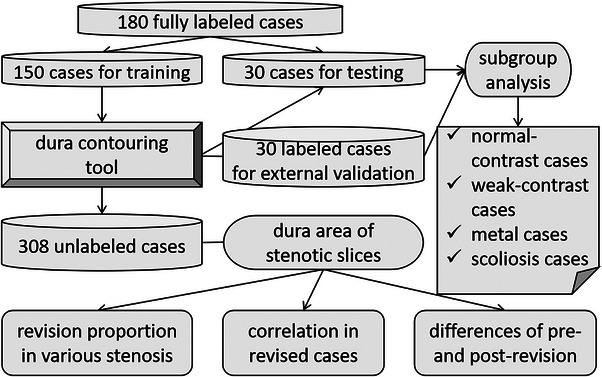
Flow diagram of the development of the dura‐contouring tool.

Of the 180 fully segmented cases, 150 cases were randomly assigned to the training and five‐fold cross‐validation dataset to train and assess the 3D U‐Net. The remaining 30 cases were regarded as the independent testing dataset to assess the segmentation performance. Another 30 fully segmented cases were used for external validation to assess the generalization ability of these DL models. The dura‐contouring tool was developed when 150 cases were used to train the 3D U‐Net, which also underwent independent testing and external validation. Finally, the dura‐contouring tool was applied to the 308 unsegmented cases for automatic segmentation and dural CSA quantification (Figure 2).

Before training the DL algorithms, all CTM images were preprocessed using the following steps: clipping (−256 to 2048), intensity normalization (−0.5–0.5), and data augmentation (flipping, noising, resizing, and patching). An initial learning rate (0.001) and Adam optimizer with drop‐out (probability of 0.25) regularization were used during the training phase. The loss function used was the SoftMax entropy loss. Additional details of the deep neural networks can be found in Figure S2. The development and assessment of the DL models were conducted on Python 3.8.13 (Python Software Foundation) with Keras 2.8.0.

### 2.4 Segmentation assessment

The Dice score (DCS) and the Hausdorff distance (HD) were used to evaluate the segmentation performance of the DL models. The DCS compares the spatial overlap of the lumbar dura for automatic segmentations with manual segmentations. The HD measures the 95th percentile distance between the automatic segmentations and the ground truth boundaries. A higher DCS and lower HD indicate better segmentation performance. Given that myelography can be complicated by factors such as metal implants, degenerative scoliosis, or stenosis‐induced contrast weakness, subgroup analyses were conducted in the independent testing and external validation datasets. The normal‐contrast cases, weak‐contrast cases, metal cases, and scoliosis cases were also assessed using both DCS and HD.

### 2.5 Second observer analysis

To further compare the segmentation performance of the dura‐contouring tool with that of human experts, a human expert (second observer) was invited to independently carry out dural segmentation in the independent testing dataset and external validation dataset. The segmentation performance of the second observer was assessed with DCS and HD based on the ground truth of the dura masks. Finally, the segmentation performance of the second observer was compared with that of the dura‐contouring tool in independent testing and external validation.

### 2.6 Application of the dura‐contouring tool

Given that the DCS and HD were indicators of the computer vision, the potential application of the dura‐contouring tool in facilitating CSA quantification of the dura still needed further validation. Thus, the dura‐contouring tool was further assessed based on how much the automatic contours needed to be edited in practice. A total of 308 unlabeled stenotic cases (446 culprit levels) were segmented with the dura‐contouring tool, which was implemented using the Keras API running on top of TensorFlow 2.8.0. The culprit level was obtained from the radiology report and subsequently subjected to further review by a spine surgeon and a pain physician. The minimum dural CSA at each stenotic level was meticulously identified and quantified.

The automatic dura masks of the stenotic slices were reviewed by three human experts, who voted on the cases that needed revision. Cases that needed revision were then manually updated by one expert, and the corresponding revisions were reviewed by the other two experts. Any disagreement would be solved by a vote of the three experts. The original automatic masks and the corresponding revised masks then generated the dural CSA on Slicer 5.0.3 (Supplemental File). The revised proportion was calculated first, but whether or how much the revision impacted the CSA of the lumbar dura remained unknown at this point. Thus, the correlation and the discrepancies in the dural CSA between the original automatic masks and the corresponding revised masks were then obtained. Additionally, subgroup analysis was conducted according to the bony canal shapes (round, oval, and trefoil).^27^

### 2.7 Statistical analysis

Statistical analysis was performed using the RStudio version 4.2.1 (The R Foundation for Statistical Computing). Continuous data are presented as the mean ± standard deviation (SD). Categorical variables were compared with the chi‐squared test or Fisher exact test, and numeric variables were compared using the student *t* test and the Mann‐Whitney test. The Pearson correlation test and Bland‐Altman analysis were conducted to assess the correlation of the dural CSA of stenotic slices between automatic masks and corresponding revised masks. The mean dural CSA was compared between the original automatic masks and the corresponding revised masks using the paired *t* test. A *p* value < 0.05 was defined as a statistically significant difference.

## 3 RESULTS

In total, 518 CTM cases were included in the study. The general data distribution is summarized in Table 1. There were no significant differences in the basic characteristics (age, gender, culprit levels, and myelography condition) among the training, independent testing, and external validation datasets (*p *> 0.05). The 3D U‐Net demonstrated favorable segmentation performance as the average DCS and HD were 0.905 ± 0.080 and 9.000 ± 14.750 mm, respectively, in the five‐fold cross‐validation (Table 2). These results excluded the occasionality of a favorable performance of the 3D U‐Net in achieving semantic segmentation of the dural sac on CTM.

**TABLE 1:** Patient characteristics in the training, validation, and testing datasets.

	Labeled cases (*n* = 210)				
Characteristic	Training (*n* = 150)	Testing (*n* = 30)	External validation (*n* = 30)	*p* value	Unlabeled cases (*n* = 308)
Age (y)	60.59 ± 15.97	61.90 ± 9.44	60.03 ± 12.36	0.844	60.30 ± 16.27
Gender				0.686	
Male	73	17	14		157
Female	77	13	16		151
Myelography				0.639	
Normal‐contrast	99	17	16		187
Weak‐contrast	23	6	7		86
Metal	18	3	4		24
Scoliosis	10	4	3		11
Culprit level				0.877	446
L1/L2	6	0	1		13
L2/L3	18	2	1		21
L3/L4	54	9	7		106
L4/L5	105	20	22		210
L5/S1	60	11	14		96

*Note*: Data are the mean ± SD and number of participants.

Abbreviation: SD, standard deviation.

**TABLE 2:** Segmentation performance of the 3D U‐Net during five‐fold cross‐validation with 150 CT myelograms.

Five‐fold cross‐validation	DCS	HD (mm)
Model 1	0.891 ± 0.122	9.787 ± 22.789
Model 2	0.932 ± 0.028	7.268 ± 12.000
Model 3	0.897 ± 0.078	12.540 ± 16.565
Model 4	0.913 ± 0.055	7.061 ± 8.224
Model 5	0.894 ± 0.118	8.344 ± 14.169
Average	0.905 ± 0.080	9.000 ± 14.750

*Note*: Data are the mean ± SD.

Abbreviations: CT, computed tomography; DCS, dice score; HD, Hausdorff distance; SD, standard deviation.

The improved performance was observed while the 150 labeled cases were directly used to train the 3D U‐Net as the dura‐contouring tool. The average DCS and HD of the dura‐contouring tool were 0.933 ± 0.018 and 4.431 ± 3.445 mm, respectively, in the independent testing, and 0.928 ± 0.034 and 5.645 ± 9.423 mm, respectively, in the external validation (Table 3). These results obtained were found to be comparable to those of the human expert in the second observer analysis. Although the average DCS of the dura‐contouring tool was slightly superior to that of the second observer both in the independent testing (0.928 ± 0.034; *p *= 0.005) and external validation datasets (0.924 ± 0.018; *p *= 0.474), the average HD of the dura‐contouring tool was slightly inferior to that of the second observer both in the independent testing (1.848 ± 0.577 mm; *p *< 0.001) and external validation datasets (2.700 ± 4.690 mm; *p *= 0.083). These results indicated that there were no absolute advantages of one method over the other in the comparisons, although statistical significance was observed during independent testing. Similarly, the subgroup analysis revealed that the DCS and HD of normal‐contrast cases, weak‐contrast cases, metal cases, and scoliosis cases were also comparable to those of the second observer (Table S1). In summary, the comparative results in the second observer analysis confirmed that a DCS of 0.92−0.93 for the dura‐contouring tool was satisfactory and acceptable.

**TABLE 3:** Segmentation performance of the contouring tool compared with the second observer in the independent testing and external validation datasets.

Comparisons	DCS	*p* value	HD (mm)	*p* value
Contour tool in independent testing	0.933 ± 0.018	0.005^*^	4.431 ± 3.445	< 0.001^*^
Second observer in independent testing	0.924 ± 0.019		1.848 ± 0.577	
Contour tool in external validation	0.928 ± 0.034	0.474	5.645 ± 9.423	0.083
Second observer in external validation	0.924 ± 0.018		2.700 ± 4.690	

*Note*: Data are the mean ± SD. *p* < 0.001, significant differences.

Abbreviations: DCS, dice score; HD, Hausdorff distance; SD, standard deviation.

**FIGURE 3:**
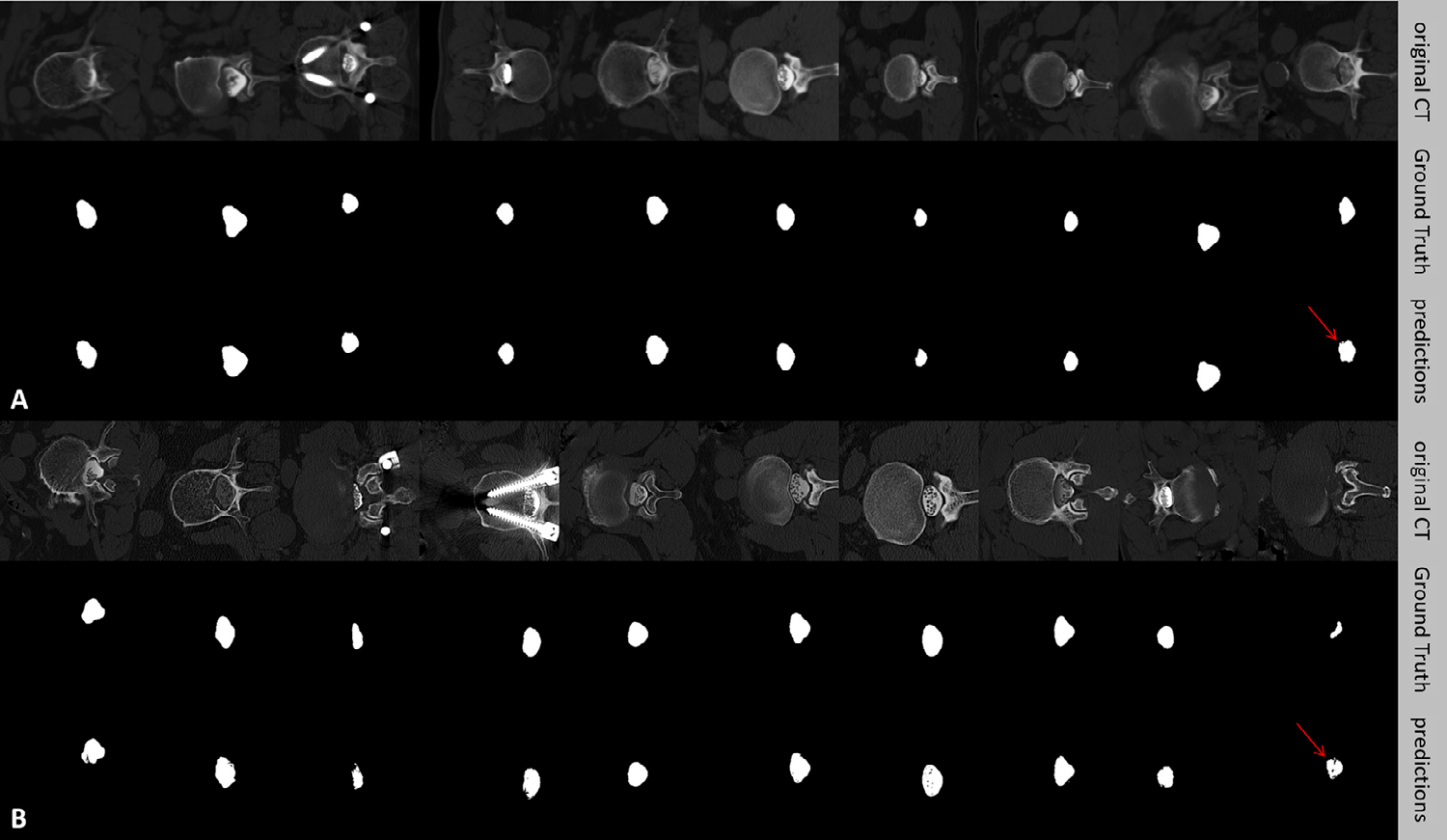
Predicted masks of the dura on CT myelogram compared with the ground truth. (a) Independent testing. (b) External validation. The red arrow represents the unsatisfactory segmentations due to the weak‐contrast cases. CT, computed tomography.

Figure 3 shows the original CTM images, the ground truth, and the predicted masks for both the independent testing and external validation datasets. These findings suggested that the dura‐contouring tool achieved generally satisfactory segmentation in most cases (i.e., normal‐contrast cases, implant cases, and scoliosis cases) but tended to overestimate the dura area in some cases (i.e., weak‐contrast cases). The dura‐contouring tool is accessible online (https://github.com/Huatsing‐Lau/CTM‐DuraSeg).

**FIGURE 4:**
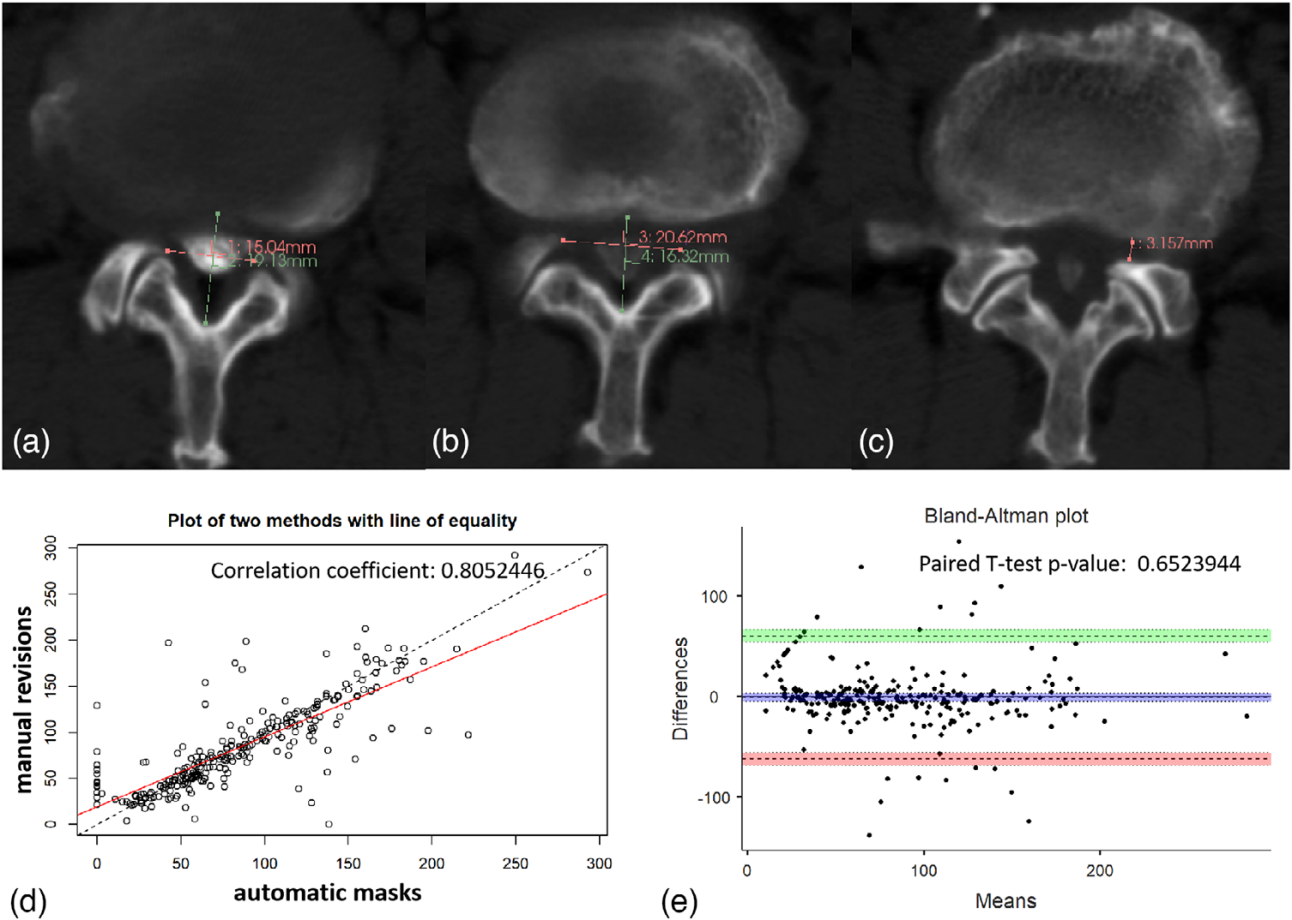
An illustration of bony canal shape and overall distribution of the dural area of the stenotic slice. (a) Round bony canal (longitudinal diameter greater than the transverse diameter). (b) Oval bony canal (transverse diameter greater than the longitudinal diameter). (c) Trefoil bony canal (longitudinal diameter of lateral recess less than 4 mm). (d) The correlation between the original automatic masks and the corresponding revised masks (*r* = 0.805). (e) The difference between the original automatic masks and the corresponding revised masks (paired *t* test, *p *= 0.652). The green and light red zones represent the 95% CI of the limits of agreement. The purple zone represents the 95% CI of the mean difference.

In the 308 unlabeled cases (Table 4), the ratio of stenotic slices requiring revisions was 58.97% (263/446). The average CSA of the original automatic masks (77.544 ± 55.785 mm^2^) was slightly larger than that of the corresponding revised masks (74.732 ± 54.849 mm2) but showed no significant difference (*p *> 0.05). For the different shapes of bony canals (Figure 4a–c), the ratio of stenotic slices needing revisions ranged from 40% to 70%, with no significant differences found between the two groups regardless of the shape of the bony canals (*p *> 0.05). The quantified dural CSA of the stenotic slice at different levels was also showed (Table 5). Significant differences in the dural CSA between the two groups were not observed, either at the upper levels (i.e., L1/L2–L3/L4) or lower levels (*p* > 0.05). These results indicated that the segmentation of the dural sac on stenotic slices with the dura‐contouring tool might need to be slightly revised in some cases, but the CSA of the stenotic dura remained broadly acceptable.

**TABLE 4:** Quantified dural area of the stenotic slice with different canal shapes.

Bony canal shape	Needed revision		Dural CSA (mm^2^)		
	Cases	Ratio	Automatic masks	Revised masks	*p* value
Average	263/446	58.97%	77.544 ± 55.785	74.732 ± 54.849	0.652
Round	33/82	40.24%	114.848 ± 51.912	105.806 ± 57.669	0.062
Oval	77/144	53.47%	107.885 ± 49.955	103.971 ± 48.739	0.158
Trefoil	153/220	69.55%	65.735 ± 43.528	68.161 ± 39.963	0.394

*Note*: Data are the numbers of participants, percentages, and the mean ± SD.

Abbreviations: CSA, cross‐sectional area; SD, standard deviation.

**TABLE 5:** Quantified dural area of the stenotic slice at different levels.

Dura area (mm^2^)	Automatic masks	Revised masks	*p* value
L1/L2 (*n* = 5)	148.315 ± 41.423	147.082 ± 43.827	0.550
L2/L3 (*n* = 8)	98.487 ± 48.426	89.429 ± 78.279	
L3/L4 (*n* = 48)	76.687 ± 44.611	66.871 ± 47.747	
L4/L5 (*n* = 131)	81.293 ± 53.249	68.691 ± 55.301	0.429
L5/S1 (*n* = 71)	89.451 ± 49.708	88.764 ± 46.872	0.765

*Note*: Data are the mean ± SD.

Abbreviation: SD, standard deviation.

**FIGURE 5:**
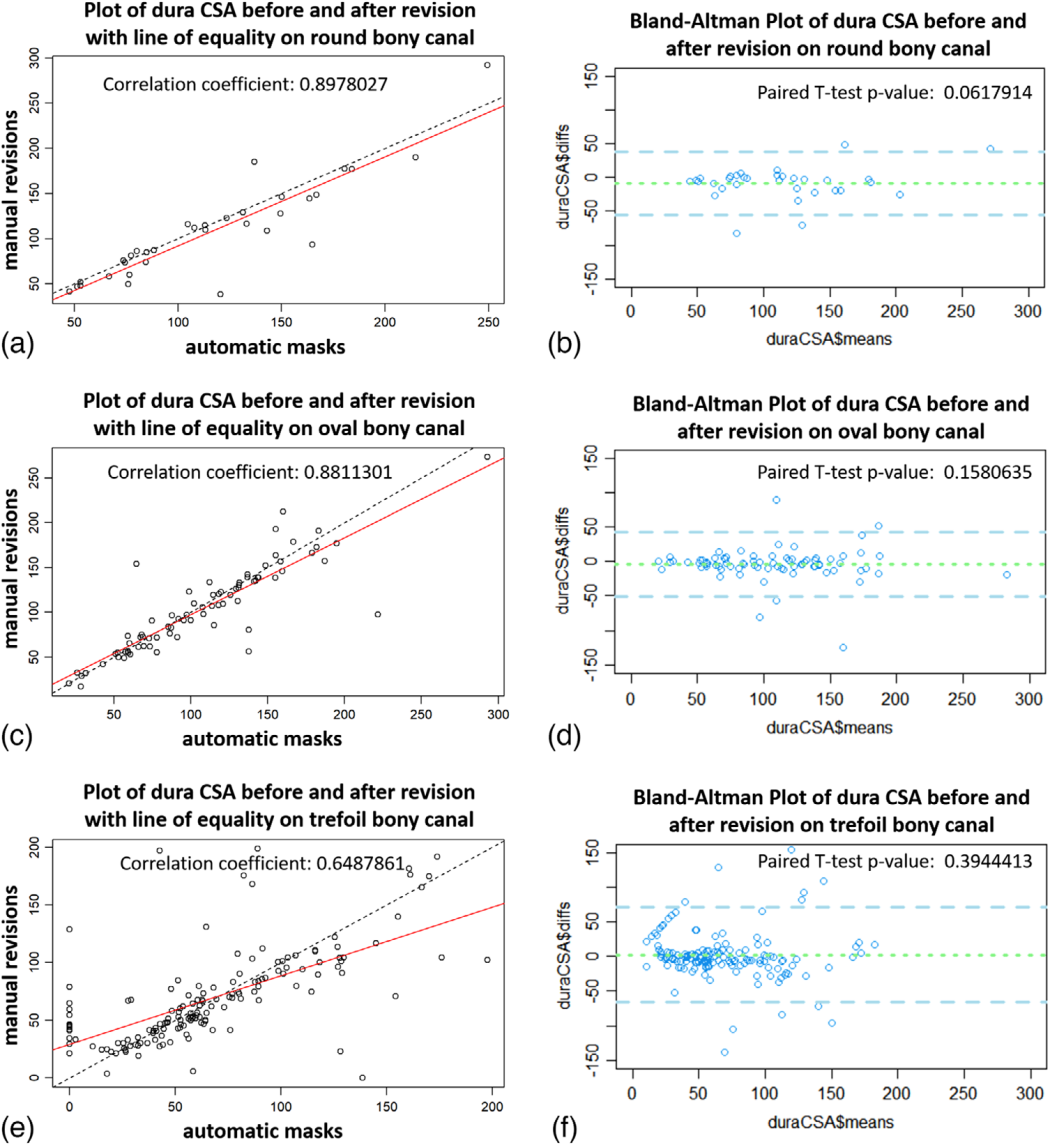
The correlation and difference of the dural area of the stenotic slice between the original automatic masks and the corresponding revised masks in different shapes of the bony canal. (a,b) Pearson correlation analysis and the Bland‐Altman plot of the round shape (*r* = 0.898; *p *= 0.06). (c,d) Pearson correlation analysis and the Bland‐Altman plot of the oval shape (*r* = 0.881; *p *= 0.158). (e,f) Pearson correlation analysis and the Bland‐Altman plot of the trefoil shape (*r* = 0.649; *p *= 0.394).

The overall relationship of the dural CSA between the original automatic masks and the corresponding revised masks is shown in Figure 4d,e. The Pearson correlation coefficient for the two groups was 0.805, and the *p* value of the paired *t* test was 0.652. Both the linear regression curve and the Bland‐Altman plot showed that the dural CSA of the automatic masks was highly consistent with those of the corresponding revised masks. Similar findings are shown in Figure 5, where the dural CSA of automatic masks was also consistent with those of corresponding revised masks independent of the bony canal shapes.

## 4 DISCUSSION

Quantitative radiologic analysis is key to the differential diagnosis and culprit localization of LSS. In this study, we have successfully developed an AI contouring tool for automatic segmentation of the lumbar dura in consecutive axial lumbar CTM slices. The AI contouring tool is capable of automatically delineating the lumbar dura with high accuracy and favorable generalization ability, efficiently, and consistently. In addition, the dura‐contouring tool also achieved decent accuracy in metal cases, scoliosis cases, and weak‐contrast cases. The automatic masks generated by the dura‐contouring tool also reliably facilitated the quantification of the dural CSA on stenotic levels regardless of the bony canal shapes. To the best of our knowledge, this is the first study to develop and disclose a dura‐contouring tool of CTM with a large sample size.

Generally, the radiologic assessment of LSS is interpreted by various imaging modalities or combinations. For example, CT scans excel in assessing bony stenosis but lack the clarity in soft tissue visualization that MRI offers. Routine MRI provides excellent soft tissue contrast, allows any slice orientation, and does not emit ionizing radiation.^28^ However, MRI still has several drawbacks, for example, the degree of stenosis can be exaggerated on T2‐weighted images.^29^, ^30^ Several studies indicated that CTM is more reliable and reproducible than MRI for preoperative evaluation of LSS patients.^31^, ^32^ While CTM is considered an older imaging modality, recent studies have highlighted its greater reliability and sensitivity in detecting multilevel stenosis cases.^33^, ^34^ Although CTM requires a lumbar puncture, making it invasive, it continues to be used and remains an essential complement in the radiological assessment for LSS patients. Myelography can reveal the details of the lumbar dura, and CT provides an excellent image of the bony canal. Therefore, CTM is effective in assessing central LSS and some lateral LSS (lateral recess stenosis), and quantitative analysis of CTM can provide additive value, which might optimize the process of radiologic evaluation and discrimination of LSS.

Recent reviews by Steurer et al.^35^ and Andreisek et al.^4^reported the qualitative and quantitative criteria for the diagnosis of LSS. Radiological parameters, such as the anteroposterior and transverse diameters of the osseous spinal canal, along with the CSA of the dural tube or sac on MR images, were used for the diagnosis of LSS. Parameters on CT images included the anteroposterior diameter of the spinal canal and dural sac, and the dural CSA. Among many proposed radiological biomarkers, quantification of the spinal canal space or the dural sac has been found to correlate with the clinical symptoms of LSS.^36^, ^37^ However, Lim et al.^12^ found that the CSA of the dural sac was more sensitive than was the CSA of the spinal canal in assessing central LSS. Thus, we opted to develop an automatic segmentation tool for the dural sac rather than the spinal canal, which would provide an efficient and consistent quantified CSA of the dural sac on CTM. However, the standards of the dural CSA that correlated to LSS were quite different among these studies.^3^ A dural area of less than 100 mm2 was reported to be considered relative stenosis at and distal to the L3/L4 level,^33^ which was also confirmed by our study. However, we also found the average dural area of stenotic slices at the L3/L4 level was less than 100 mm2 though with a small sample size. Additionally, we quantified the stenotic dura area with different shapes of the bony canal. Quantified dural CSA of stenotic slices with round/oval shapes might represent the anatomical characteristics of central LSS without lateral recess stenosis. Conversely, the dural area of the stenotic slices with a trefoil shape was much smaller, which might reflect central stenosis that was much more severe when complicated with lateral recess stenosis.

Segmentation and quantification of the dura were deemed to benefit the development of radiologic criteria.^28^, ^38^, ^39^ Moreover, automatic segmentation would eliminate interobserver variability and facilitate large‐throughput analysis pipelines.^15^ As the gray values of the dura on CTM images were similar to those of the bones, it is difficult for manual contouring to quickly distinguish the dura from the surrounding tissues using thresholds. Furthermore, a typical CTM scan often consists of dozens of 2‐dimensional image slices per patient. It is tedious and fatiguing for surgeons to delineate the dura slice by slice. Observer reproducibility and interobserver reliability are other issues of manual delineation. The segmentation accuracy highly depends on the experience of the technicians who conduct the delineation. Thus, we endeavored to achieve the automatic segmentation of the dural sac on axial CTM images with the aim to relieve technicians from tedious and cumbersome tasks. The results revealed a favorable proportion of automatic masks that needed no revisions on the culprit levels. Even among the masks needing revisions, the dural area was highly correlated, showing no significant differences between the original automatic and the corresponding revised masks on most culprit levels. These results indicated that the automatic segmentation of the dura on CTM at least enabled an acceptable and objective area quantification efficiently.

Automatic segmentation of multiple lumbar structures remains a dynamic field of study in spinal radiological assessment,^40^ including the vertebrae, intervertebral discs, lumbar dura, spinal canal, and ligaments on different imaging modalities. For example, Michopoulou et al.^41^ used an atlas‐based algorithm to achieve automatic segmentation of degenerated lumbar intervertebral discs on MRI, and the DCS of this method was 91.6% for normal discs and 87.2% for degenerated discs. Utilizing DL technique, Li et al.^22^ proposed a multiscale attention U‐shaped network for semantic segmentation of the vertebral body, lamina, and dural sac on MR images (mean DCS was 0.9257). Although multi‐atlas segmentation could potentially contour the dural sac on CTM, the use of deformable image registration in this method could significantly impair segmentation performance in cases complicated by pathological lesions (e.g., severe LSS or scoliosis).^42^ Numerous studies have confirmed the advantages of DL algorithms over the atlas‐based segmentation methods especially when segmenting cases with pathological lesions.^43^, ^44^, ^45^ Thus, we have implemented DL algorithms to develop an automatic contouring tool for the lumbar dura on CTM images. The overall segmentation performance of this dura‐contouring tool was found to be satisfactory (about 0.92 for average DCS; about 4 mm for mean HD) regardless of the myelography condition or the shape of the bony canal. The segmentation performance observed in our study was comparable with that of a second observer, which reconfirmed the potential value of DL models in LSS diagnosis and quantitative assessment.

Several limitations of this study warrant attention. First, the segmentation performance of the AI contouring tool was found to be unsatisfactory on certain CTM slices without a full filling of contrast medium (Figure 3, red arrow). However, even human experts might fail to delineate the dura on such images in a repeatable and consistent manner. Future studies with larger amounts of training data might further improve the performance of the dura‐contouring tool. Second, the culprit levels mentioned in this study were identified by radiologic assessment, not as symptomatic levels of LSS identified in clinical practice. It remains uncertain whether the dura‐contouring tool wound maintain decent segmentation performance on symptomatic stenotic levels, there should be no difference from a perspective of semantic segmentation. Third, the current study only developed an AI contouring tool for the lumbar dura, offering limited utility in assessing lateral recess stenosis and no capability in assessing neural foraminal stenosis. This limitation arises from myelography's ineffectiveness in depicting nerve roots, a challenge not rooted in technical constraint. Thus, we aim to develop a canal contouring tool in future study, which will delineate ROIs on CTM/CT/MRI and then create the basis for image feature engineering or extraction of DL features. Such advancements are expected to enhance the radiographic assessment and diagnosis of lateral LSS. Finally, the current version of the dura‐contouring tool was developed based on 3D U‐Net without comparisons with other sophisticated algorithms or the emerging promotable Transformer‐based big models. As the second observer analysis showed the dura‐contouring tool was not inferior to the human expert, we believed it was worthwhile introducing and disclosing a lightly deployable tool into the community.

## 5 CONCLUSIONS

In this paper, we propose an AI contouring tool for the automatic segmentation and quantitative analysis of the lumbar dura on CTM images. The dura‐contouring tool demonstrated high accuracy and generalization ability regardless of the myelography conditions. Additionally, the dura‐contouring tool also showed favorable application potential in patients with LSS, as it could facilitate the quantification of the dural area on stenotic levels regardless of the shape of the bony canal.

## AUTHOR CONTRIBUTIONS

All authors contributed to the study conception and design. Material preparation, data collection, model development and data analysis were performed by Guoxin Fan, Dongdong Wang, Jianjin Zhang, Huaqing Liu, Yufeng Li, and Xiaokang Du. Huaqing Liu and Xiang Liao was involved in study conception, design and program supervision. The first draft of the manuscript was written by Dongdong Wang and Guoxin Fan, and all authors commented on previous versions of the manuscript. All authors read and approved the final manuscript.

## CONFLICT OF INTEREST STATEMENT

The authors declared that they have no competing interests as defined by **Journal of Applied Clinical Medical Physics**, or other interests that might be perceived to influence the results and/or discussion reported in this paper.

## ETHICS APPROVAL AND CONSENT TO PARTICIPATE

The study was conducted in accordance with the Declaration of Helsinki and approved by the institutional ethical committees of Huazhong University of Science and Technology Union Shenzhen Hospital ([2020]02‐252‐01), and Third Affiliated Hospital, Sun Yat‐Sen University (KY‐2022‐031‐01). Written patient consent was waived due to the retrospective nature of the study, and all data were deidentified. The need for informed consent was waived by the Ethical Committees of Huazhong University of Science and Technology Union Shenzhen Hospital and Third Affiliated Hospital, Sun Yat‐Sen University.

## CONSENT FOR PUBLICATION

Not applicable.

## Supporting information

Supporting Information

Supporting Information

## Data Availability

The original contributions presented in the study are included in the article/Supplementary Materials, further inquiries can be directed to the corresponding authors. The dura‐contouring tool is available online (https://github.com/Huatsing‐Lau/CTM‐DuraSeg). The data that support the findings of this study are available from the corresponding author upon reasonable request.
