# Supplementary material for: Automatic segmentation of dura for quantitative analysis of lumbar stenosis: A deep learning study with 518 CT myelograms
Source: J Appl Clin Med Phys. 2024 May 10;25(7):e14378. doi: 10.1002/acm2.14378 (PMC11244674; doi:10.1002/acm2.14378)
Supplement: Supplementary file 1 — Supporting Information [file ACM2-25-e14378-s001.docx]

**Supplemental Table 1. Segmentation performance of the dura-contouring tool compared with a second observer in different myelography conditions**

| **Comparison** | **Myelography** | **DCS** | **HD (mm)** |
| --- | --- | --- | --- |
| **Contour tool in independent testing** | **All (*n* = 30)** | 0.933 ± 0.018 | 4.431 ± 3.445 |
|  | **Normal-contrast (*n* = 17)** | 0.934 ± 0.019 | 4.896 ± 3.603 |
|  | **Weak-contrast (*n* = 6)** | 0.918 ± 0.017 | 4.997 ± 3.527 |
|  | **Scoliosis (*n* = 4)** | 0.925 ± 0.020 | 3.781 ± 3.100 |
|  | **Metal (*n* = 3)** | 0.941 ± 0.003 | 1.727 ± 0.725 |
| **Second observer in independent testing** | **All (*n* = 30)** | 0.924 ± 0.019 | 1.848 ± 0.577 |
|  | **Normal-contrast (*n* = 17)** | 0.930 ± 0.013 | 0.580 ± 0.142 |
|  | **Weak-contrast (*n* = 6)** | 0.908 ± 0.024 | 0.700 ± 0.193 |
|  | **Scoliosis (*n* = 4)** | 0.925 ± 0.023 | 1.692 ± 0.629 |
|  | **Metal (*n* = 3)** | 0.912 ± 0.013 | 2.187 ± 0.572 |
| **Contour tool in external validation** | **All (*n* = 30)** | 0.928 ± 0.034 | 5.645 ± 9.423 |
|  | **Normal-contrast (*n* = 16)** | 0.942 ± 0.016 | 3.459 ± 2.444 |
|  | **Weak-contrast (*n* = 7)** | 0.906 ± 0.056 | 13.468 ± 17.876 |
|  | **Scoliosis (*n* = 3)** | 0.894 ± 0.021 | 2.183 ± 0.559 |
|  | **Metal (*n* = 4)** | 0.933 ± 0.015 | 3.667 ± 1.155 |
| **Second observer in external validation** | **All (*n* = 30)** | 0.924 ± 0.018 | 2.700 ± 4.690 |
|  | **Normal-contrast (*n* = 16)** | 0.933 ± 0.011 | 1.464 ± 1.065 |
|  | **Weak-contrast (*n* = 7)** | 0.909 ± 0.025 | 6.728 ± 8.831 |
|  | **Scoliosis (*n* = 3)** | 0.929 ± 0.012 | 2.011 ± 1.302 |
|  | **Metal (*n* = 4)** | 0.913 ± 0.009 | 1.111 ± 0.154 |

Data are the mean ± SD. DCS, Dice score; HD, Hausdorff distance.
