# Supplementary material for: Automatic segmentation of dura for quantitative analysis of lumbar stenosis: A deep learning study with 518 CT myelograms
Source: J Appl Clin Med Phys. 2024 May 10;25(7):e14378. doi: 10.1002/acm2.14378 (PMC11244674; doi:10.1002/acm2.14378)

# Supplemental file

**Figure legends of Supplemental figures:**

**Supplemental Figure 1**. CTM data reconstructed with different algorithms. (A) Data reconstructed with the soft-tissue algorithm for the training and testing datasets. (B) Data reconstructed with the bone algorithm for the external validation dataset. CTM, computed tomography myelogram.

**Supplemental Figure 2**. Details of the deep neural networks.

**CSA calculation with the 3D Slicer.**

1. The dura mask was cropped on the stenotic slice.
2. Go to “Segment Editor” module
3. Select scissors tool
4. Select “Erase outside”
5. Select “Rectangle”
6. Drag the cursor to the target slice and then release


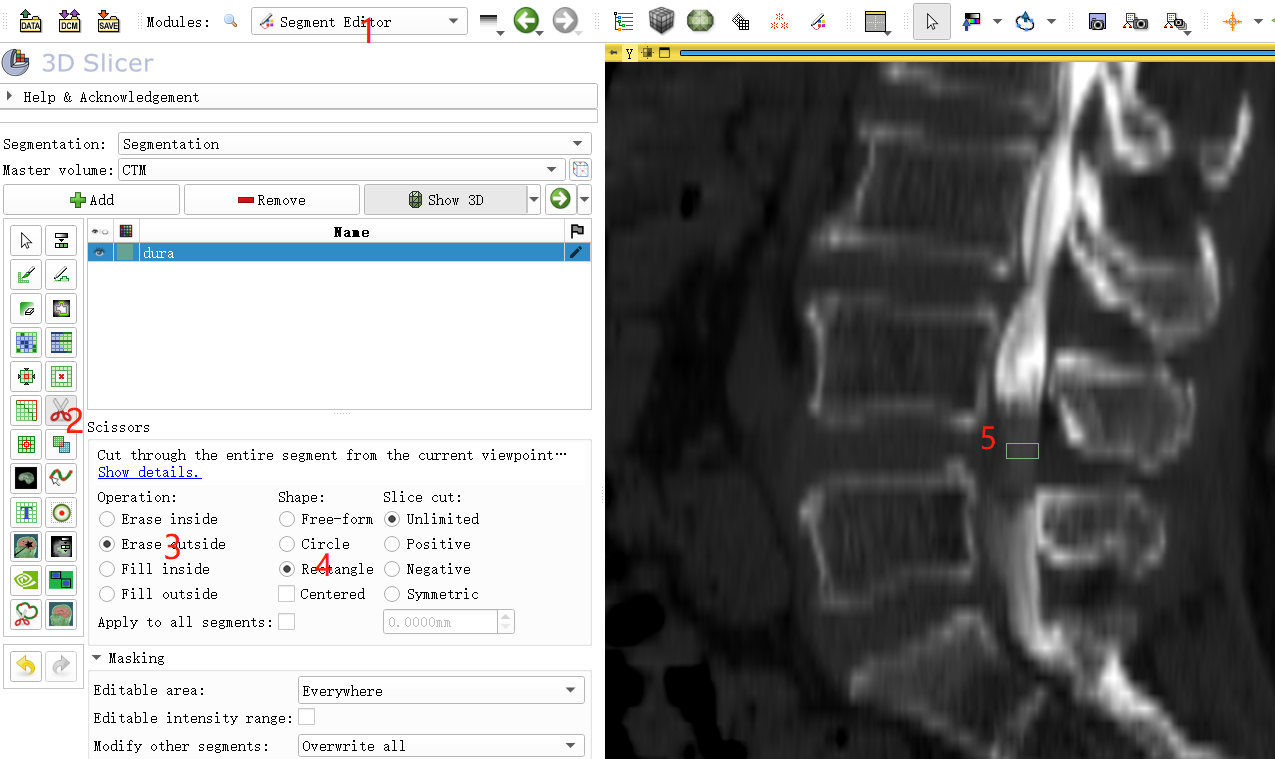


1. The volume of this mask was computed.

(1) Go to “Segmentations” module

(2) Select “Models”

(3) Select “Export”


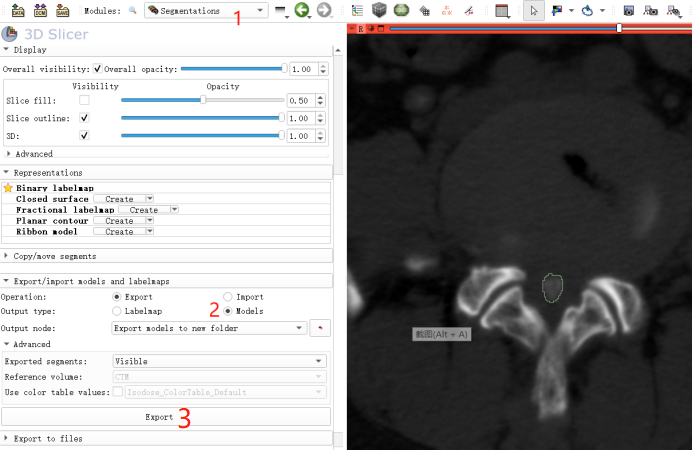


1. The CSA was obtained when the volume of this mask was divided by the slice thickness of this case.


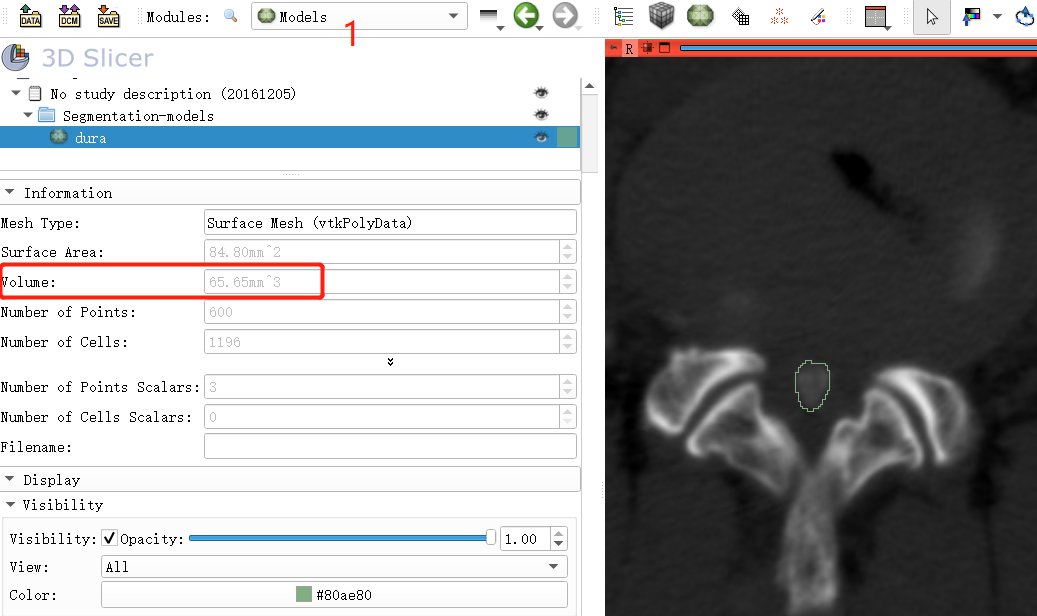


(1) Go to “Models” module

(2) Record “Volume”


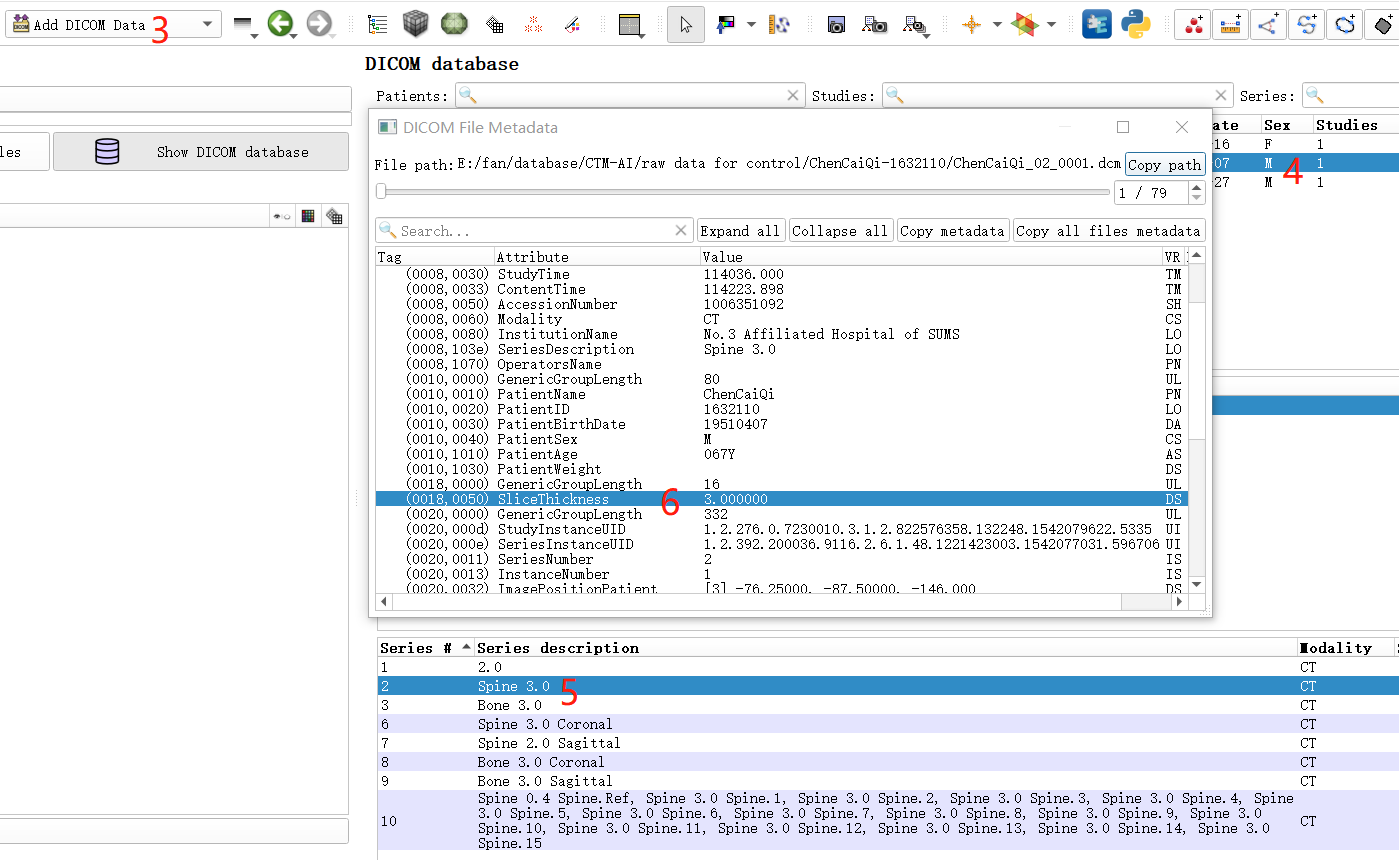


(3) Go to “Add DICOM Data” module

(4) Select the target case

(5) Select the volume and then right-click the mouse, select “View DICOM metadata”

(6) Record the slice thickness, and finally calculate the CSA = volume/slice thickness

CSA, cross-sectional area.

**Details of the hyperparameter settings**

The hyperparameter settings of the training 3D U-Net were as follows:

img_channels: 1

batch_size:8

learning_rate: 0.001

weights', default=[1, 4],

loss = weighted_softmax_cross_entropy_loss(preds, labels, weights, n_cls)

optimizer = tf.compat.v1.train.AdamOptimizer(learning_rate=learning_rate,

beta1=0.9, beta2=0.999,

epsilon=1e-08,

use_locking=False,

name='Adam').minimize(loss)

## Preprocessing details


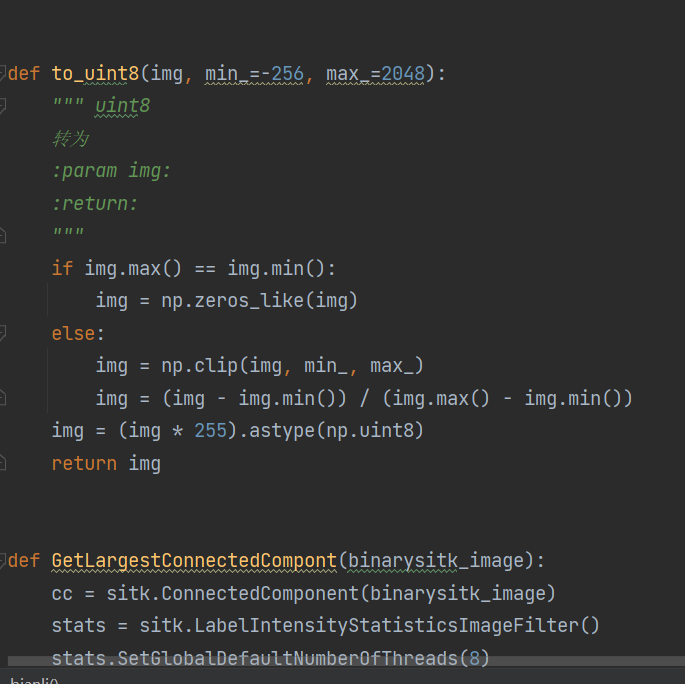


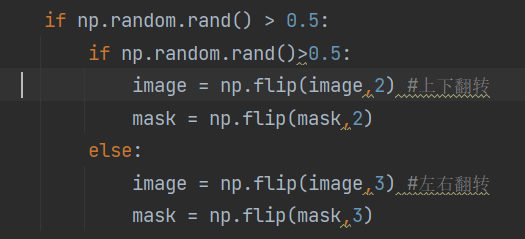


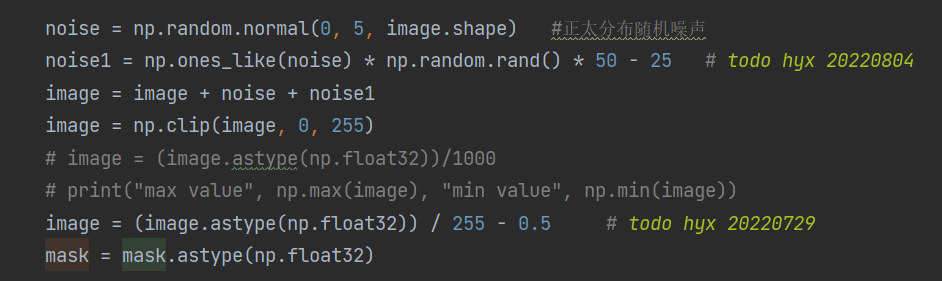


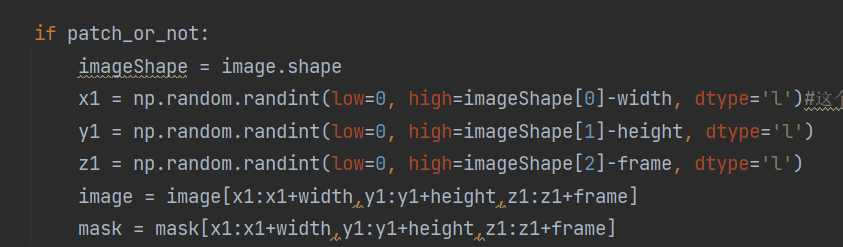


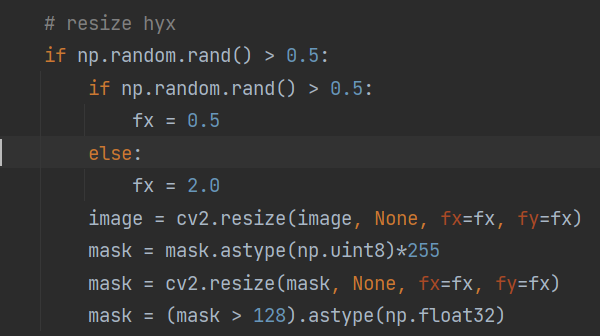

Supplement: Supplementary file 2 — Supporting Information [file ACM2-25-e14378-s002.docx]
